# The First Case of Bacillus Calmette‐Guérin‐Induced Autoimmune Encephalitis

**DOI:** 10.1002/iju5.70134

**Published:** 2026-01-04

**Authors:** Makoto Ishii, Haruto Honda, Seigo Machiya, Hiromu Horitani, Sayaka Horii, Masao Kobayashi, Yutaka Ono

**Affiliations:** ^1^ Department of Urology Higashiosaka City Medical Center Higashiosaka City Osaka Japan

**Keywords:** autoimmune encephalitis, BCG‐related side effect, intravesical BCG therapy, plasmapheresis, steroid pulse

## Abstract

**Introduction:**

Intravesical bacillus Calmette‐Guerin (BCG) therapy is a standard treatment for intermediate to high‐risk non‐muscle invasive bladder cancer. While local side effects are common, systemic complications are rare and can be serious.

**Case Presentation:**

A 69‐year‐old man presented with episodes of fever, headache, and impaired consciousness. He had undergone intravesical BCG therapy 3 days before presentation. He was initially diagnosed with viral encephalitis and treated with acyclovir, but showed no clinical improvement. Given the lack of response, autoimmune encephalitis was suspected, and steroid therapy was initiated, resulting in marked clinical improvement. Plasmapheresis was performed in addition to steroid therapy, and his condition improved to the level observed prior to BCG treatment. To date, he remains stable and relapse‐free.

**Conclusion:**

To our knowledge, this is the first reported case of BCG‐induced autoimmune encephalitis after intravesical therapy.

## Keynote Message

We report a rare case of autoimmune encephalitis after intravesical BCG therapy. The case highlights a potentially overlooked neurological adverse event that may be associated with this therapy. This report may contribute to the differential diagnosis of fever and impaired consciousness after BCG instillation.

## 1 Introduction

We present an unrecognized neurological complication of bacillus Calmette‐Guerin (BCG) therapy for non‐muscle invasive bladder cancer [1]. Intravesical instillation of live BCG is recommended for intermediate to high‐risk non‐muscle invasive bladder cancer, but some patients suffer toxicity and serious adverse events. We report autoimmune encephalitis, a rare adverse event related to BCG intravesical instillation therapy.

## 2 Case Presentation

A 69‐year‐old man presented at the emergency department with episodes of fever, headache, and impaired consciousness. He was treated with transurethral bladder tumor resection for intravesical tumor recurrence after surgery for right ureteral cancer. He was then treated with BCG intravesical instillation because the pathology result was carcinoma in situ. Three days after his fourth and last BCG treatment, the patient developed reactive arthritis, and steroid treatment (prednisolone 20 mg daily) was started. One month after the start of steroid treatment, he presented with the aforementioned complaints.

**FIGURE 1:**
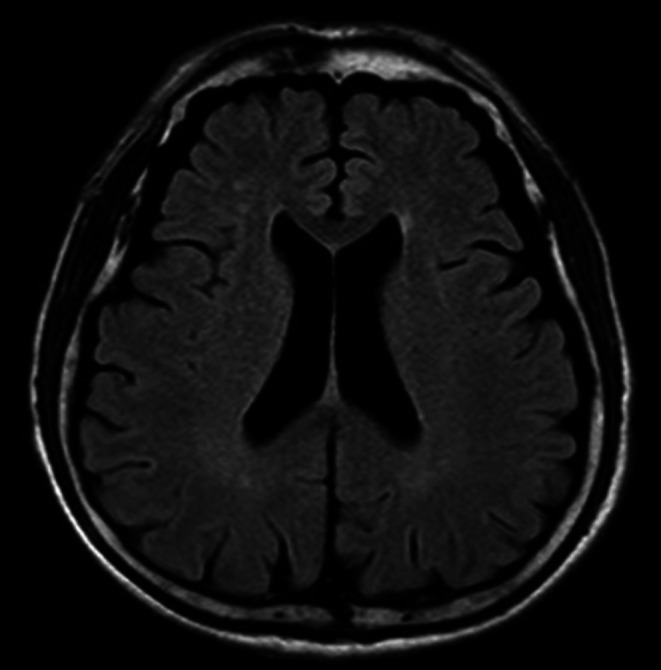
Magnetic resonance imaging. No definite areas of abnormal high signal intensity are identified on diffusion‐weighted imaging. There are no high signal lesions on T2‐weighted or FLAIR images suggestive of encephalitis. No obvious intracranial hemorrhage or gross cerebral infarction is observed.

**FIGURE 2:**
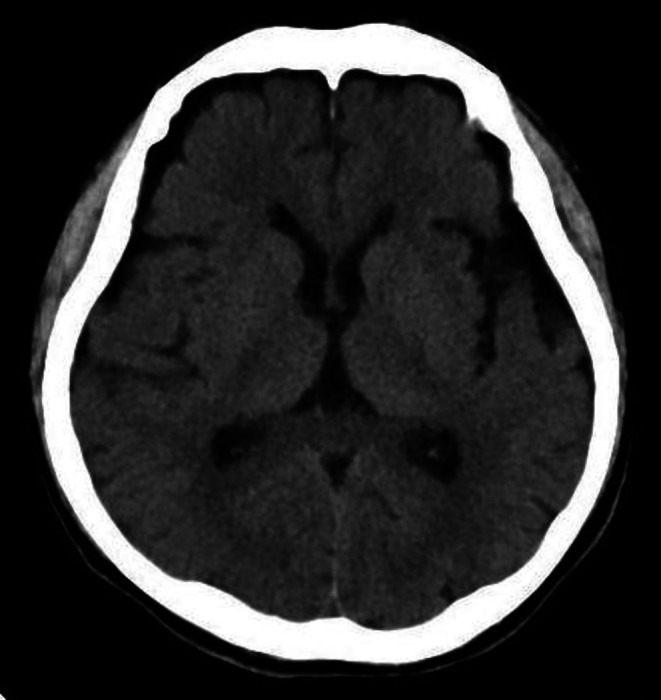
Computed tomography. No obvious pathological findings are observed.

The patient's level of consciousness was GCS E4V3M6, with mild memory impairment and disorientation. He had no rigidity of the neck. At the time of presentation, vital signs were stable with the exception of fever. Blood work revealed a normal white blood cell count (7400/μL), slightly low hemoglobin (9.2 g/dL), normal platelets (222 × 10^3^/μL), slightly high creatinine (1.24 mg/dL), slightly high C‐reactive protein (1.41 mg/dL), and normal serum sodium, potassium, calcium, and coagulation parameters. Cerebrospinal fluid (CSF) analysis showed an elevated cell count of 15.33/μL (normal range 0–5 μL), with a mononuclear cell predominance (93%), a normal glucose level of 70 mg/dL (normal range 40–75 mg/dL), and a slightly high protein level of 53 mg/dL (normal range 10–40 mg/dL). Head computed tomography (Figure 1) and magnetic resonance imaging (MRI) (Figure 2) were unchanged. Electroencephalography showed no abnormalities.

Based on the above‐described observations, encephalitis due to BCG infection, autoimmune encephalitis, or viral encephalitis was suspected. BCG‐induced encephalitis was excluded because there was no evidence of generalized infection, and CSF analysis showed a mildly elevated cell count with a mononuclear cell predominance. Because the patient was receiving steroids for reactive arthritis (prednisolone 15 mg daily), we considered viral encephalitis more likely than autoimmune encephalitis and started treatment with acyclovir.

Acyclovir was started on the day of admission, but on the third day, the patient developed a fever in the 39°C range, his level of consciousness decreased to GCS E2V3M5, and nuchal rigidity appeared. Lumbar puncture was performed again, and the analysis results were the same as those on admission, and viral cultures and herpes virus PCR were also negative.

**FIGURE 3:**
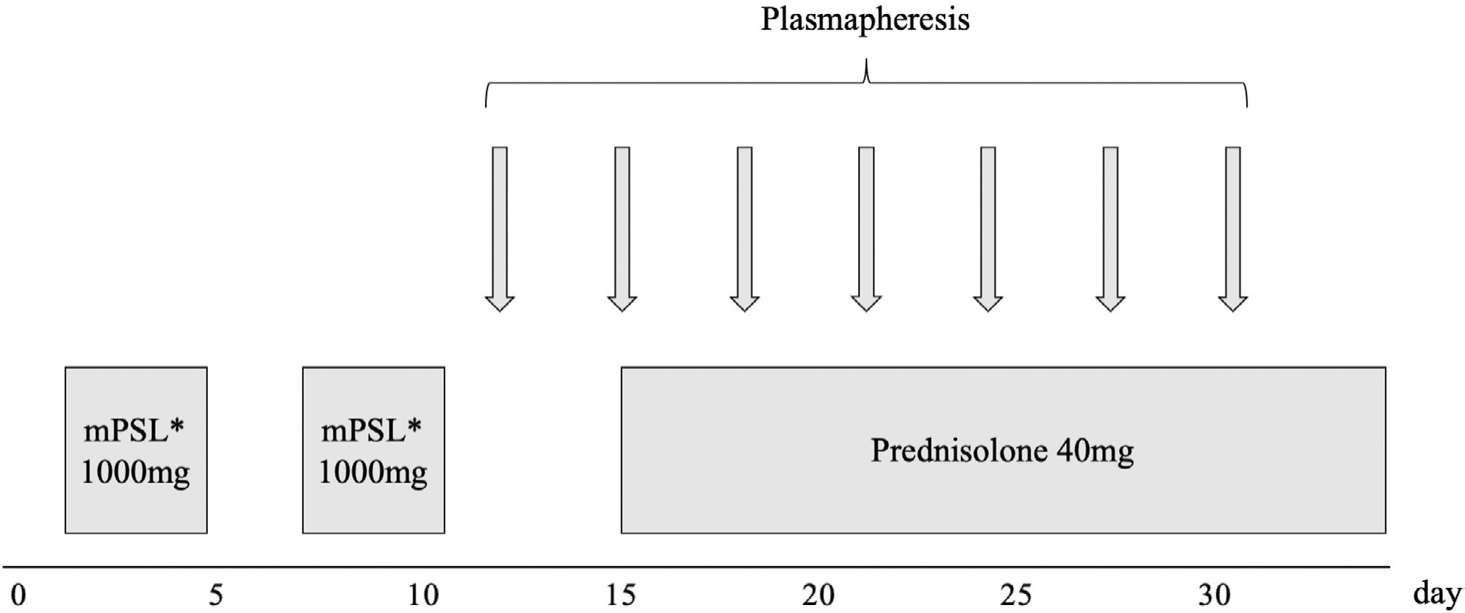
Clinical course of steroid treatment and plasmapheresis. *mPSL, methylprednisolone.

At this point, we determined that the patient was experiencing an exacerbation of autoimmune encephalitis rather than viral encephalitis and initiated steroid pulse therapy (Figure 3). After the administration of steroid pulses, he exhibited reduced fever, and the symptoms of nuchal rigidity improved. After some time, there were intermittent episodes of reduced consciousness. Therefore, another round of steroid pulse therapy was administered. At this stage, CSF culture, including for acid‐fast bacilli, and blood and urine cultures were negative. CSF PCR testing was also negative. Given these results, we surmised that a diagnosis of autoimmune encephalitis was correct and continued treatment accordingly.

Seven sessions of plasmapheresis were subsequently performed (Figure 3), and the patient has now recovered to almost his pre‐onset condition. With respect to steroids, prednisolone was tapered from 40 mg and is currently being continued at a dose of 1 mg. To date, the patient remains stable and relapse‐free.

## 3 Discussion

Although BCG is the standard treatment for superficial bladder cancer, there is substantial concern about potential associated toxicity, either local or systemic, and those adverse effects remain the main limitation against its use. Local adverse effects include bladder irritation, hematuria, pyuria, and bladder atrophy. Systemic adverse effects reported include fever, reactive arthritis (conjunctivitis, urethritis, and arthritis), disseminated BCG infection, and interstitial pneumonia [2].

Intravesical immunotherapy with BCG was first reported by Molares et al. [3], who noted that instilling large amounts of live BCG bacteria into the bladder facilitated effective BCG treatment of superficial bladder cancer in 1976. The mechanism of the antitumor effects of intravesical BCG therapy is not yet fully understood, but it is believed to involve elements of nonspecific inflammation, such as desquamation of the bladder mucosa, natural immunity through effectors such as natural killer cells, and the involvement of acquired immunity, primarily through T cells [4]. In the course of these reactions to BCG, the hypersensitivity reaction is thought to activate autoimmunity and cause immune‐related adverse events (irAEs).

A review was conducted to investigate reports of irAEs associated with intravesical BCG therapy, such as the present case. According to Peng et al. [5] a total of 871 cases of irAEs have been recorded in the FAERS database, spanning 2004 to 2023, of which 17 involved nervous system toxicities. However, there were no reports of autoimmune encephalitis.

In cases of immune‐related autoimmune encephalitis, early diagnosis is crucial because immunotherapy can be highly effective; however, making an early clinical diagnosis is often challenging. In the present case, the diagnosis was made using the newly proposed criteria by Graus et al. [6], which state that a diagnosis can be made when all three of the following criteria are fulfilled: First, there must be a subacute onset of working memory deficits, altered mental status, or psychiatric symptoms. Second, at least one of the following findings must be present: new focal central nervous system findings; seizures not explained by a previously known seizure disorder; CSF pleocytosis; or MRI features suggestive of encephalitis. Finally, alternative causes must be reasonably excluded.

In the present case, the patient met the first two criteria based on the presence of memory impairment, disorientation, and elevated CSF cell counts. Moreover, potential alternative etiologies such as central nervous system infections, metabolic encephalopathy, cerebrovascular disease, and neoplastic disorders were excluded, allowing for a diagnosis of autoimmune encephalitis. The marked response to steroid therapy further supported this diagnosis.

The potential relationship between reactive arthritis and autoimmune encephalitis remains open to discussion. To date, no studies have directly linked these two disorders. However, as previously noted, although rare, several neurological complications have been reported following intravesical BCG therapy [5]. Given that BCG instillation can induce reactive arthritis through immune activation, it is plausible that similar immunological mechanisms—particularly T‐cell–mediated responses and heightened systemic immune activation—may contribute to central nervous system autoimmunity in susceptible individuals. Both conditions share a common immunological background characterized by post‐infectious or immune‐triggered inflammatory responses. Therefore, in the present case, BCG‐induced immune activation may have promoted autoimmune processes within the central nervous system, ultimately leading to the development of autoimmune encephalitis.

This is the first reported case of BCG‐induced autoimmune encephalitis after intravesical therapy. Although such occurrences are rare, as demonstrated in the present case, intravesical BCG therapy can lead to serious side effects, and careful follow‐up is required.

## Ethics Statement

The authors have nothing to report.

## Consent

The authors have nothing to report.

## Conflicts of Interest

The authors declare no conflicts of interest.

## Data Availability

The data that support the findings of this study are available on request from the corresponding author. The data are not publicly available due to privacy or ethical restrictions.
